# Different copies of SENSITIVITY TO RED LIGHT REDUCED 1 show strong subfunctionalization in *Brassica napus*

**DOI:** 10.1186/s12870-019-1973-x

**Published:** 2019-08-22

**Authors:** Sarah Schiessl, Natalie Williams, Pascal Specht, Dorothee Staiger, Mikael Johansson

**Affiliations:** 1 RNA Biology and Molecular Physiology, Faculty for Biology, Bielefeld University, Universitaetsstrasse 25, 33615 Bielefeld, Germany; 2 Department of Plant Breeding, Justus Liebig University, IFZ Research Centre for Biosystems, Land Use and Nutrition, Heinrich-Buff-Ring 26-32, 35392 Giessen, Giessen, Germany

**Keywords:** *Arabidopsis*, *Brassica napus*, Flowering, Cross-species functionality, Subfunctionalization

## Abstract

**Background:**

Correct timing of flowering is critical for plants to produce enough viable offspring. In *Arabidopsis thaliana* (Arabidopsis), flowering time is regulated by an intricate network of molecular signaling pathways. Arabidopsis *srr1–1* mutants lacking SENSITIVITY TO RED LIGHT REDUCED 1 (SRR1) expression flower early, particularly under short day (SD) conditions (1). SRR1 ensures that plants do not flower prematurely in such non-inductive conditions by controlling repression of the key florigen *FT*. Here, we have examined the role of SRR1 in the closely related crop species *Brassica napus.*

**Results:**

Arabidopsis SRR1 has five homologs in *Brassica napus.* They can be divided into two groups, where the A02 and C02 copies show high similarity to AtSRR1 on the protein level. The other group, including the A03, A10 and C09 copies all carry a larger deletion in the amino acid sequence. Three of the homologs are expressed at detectable levels: *A02*, *C02* and *C09*. Notably, the gene copies show a differential expression pattern between spring and winter type accessions of *B. napus*. When the three expressed gene copies were introduced into the *srr1–1* background, only *A02* and *C02* were able to complement the *srr1–1* early flowering phenotype, while *C09* could not. Transcriptional analysis of known SRR1 targets in *Bna.SRR1*-transformed lines showed that *CYCLING DOF FACTOR 1 (CDF1)* expression is key for flowering time control via SRR1.

**Conclusions:**

We observed subfunctionalization of the *B. napus SRR1* gene copies, with differential expression between early and late flowering accessions of some *Bna.SRR1* copies. This suggests involvement of *Bna*.*SRR1* in regulation of seasonal flowering in *B. napus*. The *C09* gene copy was unable to complement *srr1–1* plants, but is highly expressed in *B. napus*, suggesting specialization of a particular function. Furthermore, the C09 protein carries a deletion which may pinpoint a key region of the SRR1 protein potentially important for its molecular function. This is important evidence of functional domain annotation in the highly conserved but unique SRR1 amino acid sequence.

**Electronic supplementary material:**

The online version of this article (10.1186/s12870-019-1973-x) contains supplementary material, which is available to authorized users.

## Background

Plants need to synchronize their reproductive activity to the optimal growth season, to ensure maximal reproductive output. Consequently, onset of flowering is tightly controlled by a network of signals originating from developmental, as well as environmental signaling pathways [2–4]. After reaching a critical developmental age, plants will respond to favorable environmental stimuli and flowering will be initiated [5]. In long day (LD) plants, flowering is promoted in spring and summer when the days are longer than the nights. Day length is measured by the inner circadian clock that maintains a ca 24-h cyclic rhythm of gene and protein expression of clock components that in turn regulate downstream processes. When light coincides with the expression of components of the so-called photoperiodic pathway of flower induction, expression of “florigen” *FLOWERING LOCUS T (FT)* is promoted in the leaves [6, 7]. FT protein then travels through the vasculature to the shoot apex where flower formation is initiated [8–10]. CONSTANS (CO) is a key signal integrator for photoperiodic flowering. Its transcription is controlled by the circadian clock through the GIGANTEA (GI) clock component that interacts with FLAVIN BINDING, KELCH REPEAT, F-Box 1 (FKF1) in coincidence with light. FKF1 then represses the activity of CDF transcription factors, which have a repressive role on *CO* expression [11–13]. This allows accumulation of *CO* transcript in the afternoon and CO protein expression. CO in turn promotes expression of *FT* by binding to its promoter and thus initiating flowering. Transcription of *FT* is also tightly regulated by both promotive and repressive elements that integrate signals from various environmental and developmental signaling pathways [2]. An important *FT* repressor in this transcriptional landscape is the MADS box transcription factor FLOWERING LOCUS C (FLC)*,* which has an important role as a repressor of flowering in unfavorable conditions, as its expression level is reduced by extended periods of cold [14, 15].

The main genetic factors of the flowering time regulation network have been conserved throughout *Brassicaceae*, as revealed by genome sequencing in recent years [16–20]. This conservation indicates that their function might be similar as in the model species Arabidopsis. Additionally, many quantitative trait loci (QTL) studies and genome-wide association studies (GWAS) for flowering time have found homologs of Arabidopsis flowering time genes in the confidence intervals of associated markers [21–29]. However, the most important crop plants from the *Brassicaceae* come from the genus *Brassica*, including important vegetable species like cabbage, cauliflower (*Brassica oleracea*), Chinese cabbage (*Brassica rapa*), but also the important oilseed crop rapeseed (*Brassica napus*). *Brassica* species share a whole-genome triplication, and *B. napus* arose from a recent interspecific hybridization between *B. rapa* (A subgenome donor) and *B. oleracea* (C subgenome donor), expanding the theoretically expected copy number of Arabidopsis homologs in allotetraploid *B. napus* to 6 (Brassica triplication × 3, hybridization × 2) [30, 31]. After polyploidization, many different processes like homologous recombination and the action of transposable elements led to a strong genome reorganization. Together with selective processes, this reorganization individually changed the specific gene copy numbers, now varying between 1 and 12, and possibly varying between individuals [16, 32, 33]. In the course of evolution, single copies might evolve differently and give rise to new expression patterns or functions through a process called subfunctionalization [34]. The degree of subfunctionalization is gene specific. Subfunctionalization has played an important role in evolution of flowering time control [35, 36].

SENSITIVITY TO RED LIGHT REDUCED (SRR1) is essential for repression of flowering in non-inductive photoperiods in Arabidopsis [1]. Mutant *srr1–1* plants flower particularly early under SD conditions and show a reduced sensitivity to the lengthening of the photoperiod. SRR1 acts to promote the expression of several direct repressors of *FT*, including *CDF1,* the *TEMPRANILLO (TEM)* transcription factors that are also involved in gibberellic acid biosynthesis and *FLC*, ensuring that flowering is prevented in non-inductive conditions. In addition, SRR1 has roles in setting the correct pace of the circadian clock and in mediating red light signaling [37]. SRR1 was also found to be important for control of flowering time in natural conditions, together with many genes closely associated to the circadian clock in a combined genome-wide association (GWAS) and linkage mapping study in Arabidopsis [38]. The protein structure of SRR1 is unknown and it does not contain any known protein motifs, although it is highly conserved between species, with homologs present in yeast and mammals [37, 39]. In *Brassica rapa*, a quantitative trait loci (QTL) study combining whole genome transcript variation with flowering time QTLs, identified the *BrSRR1* ortholog as a candidate associated with flowering and the expression of *BrFT* [40]. Furthermore, the *Bna.SRR1.A02* copy has recently been identified as one of the candidates genes responsible for the morphotypic split between biannual and annual forms in *B. napus* [41]. This suggests that the role for SRR1 in flowering time control may be conserved among *Brassicaceae*.

*B. napus* carries 5 copies of *Bna.SRR1* located on chromosomes *A02*, *A03*, *A10*, *C02* and *C09*. It is unclear if all of them have maintained the original function or if they have undergone subfunctionalization processes. Here, we examine the functionality of the *Bna.SRR1* copies by expression analysis in *B. napus* and complementation of Arabidopsis *srr1–1* mutants. We show that two groups of different gene structures have evolved and that only some *Bna.SRR1* gene copies are functional in Arabidopsis. This indicates a strong subfunctionalization of *Bna.SRR1* and provides new information about SRR1 function.

## Results

### Phylogeny of SRR1 in Brassicaceae

**Fig. 1:**
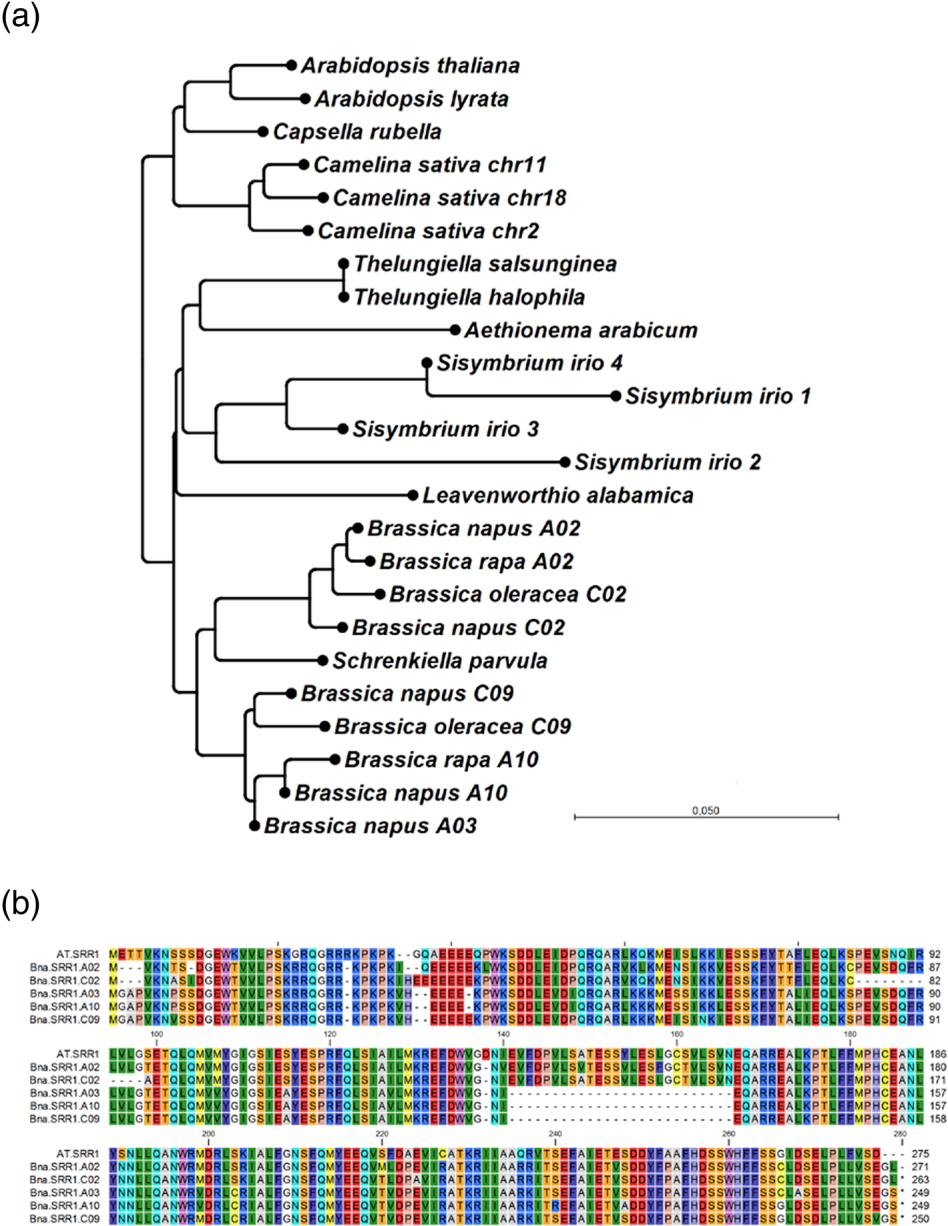
**a** Neighbor-joining tree for predicted protein sequences of SRR1 copies in 13 different species of the *Brassicaceae*. Genomic sequences were extracted from BRAD. Sequence alignment was performed using CLUSTAL multiple sequence alignment by MUSCLE with Default parameters. Based on this alignment, neighbor joining tree using bootstrap analysis (100 replicates) was constructed using CLCSequenceViewer, version 8.0. **b** Full length alignment of the predicted amino acid sequences of *At.SRR1* with the 5 *Bna.SRR1* copies

We searched 13 sequenced *Brassicaceae* species for homologs of *A. thaliana SRR1*. Copies of *SRR1* were found in all 13 species (Fig. 1a). Most of them (8 out of 13, *A. thaliana, A. lyrata, Capsella rubella, Thelungiella salsunginea, Thelungiella halophila, Aethionema arabicum, Leavenworthia alabamicum, Schrenkiella parvula*) carried only one copy of *SRR1*, whereas *B. rapa* and *B.oleracea* each carried two copies, *Camelina sativa* carried three copies, *Sisymbrium irio* four copies and *B. napus* five copies. Thus, *B. napus* carries one copy more than expected from its progenitor species. Sequence comparisons indicate that the *Bna.SRR1.A03* copy arose from a duplication of the *Bna.SRR1.A10* copy (Fig. 1a).

Gene sequence analysis shows that the five *Bna.SRR1* copies can be divided into two groups, based on their predicted amino acid sequence. The first group, consisting of the *A02* and *C02* gene copies, is more similar to the AtSRR1 protein although several amino acid changes have occurred (Fig. 1b). The second group, consisting of the *A03*, *A10* and *C09* gene copies, all have a 21 amino acid deletion in their protein sequences, compared to the AtSRR1 protein and the A02 and C02 proteins (similarity to AtSRR1: A02: 83.6% and C02: 80.7% conservation vs A03: 73.4%, A10: 73.8% and C09 74.9% conservation). Only one copy in *B. rapa* and *B.oleracea* and two copies in *S. irio* showed similar deletions in this region. A 13 amino acid deletion is also found in the C02 protein, which is unique for this homolog (Fig. 1b).

### Not all *Bna.SRR1* copies are expressed

**Fig. 2:**
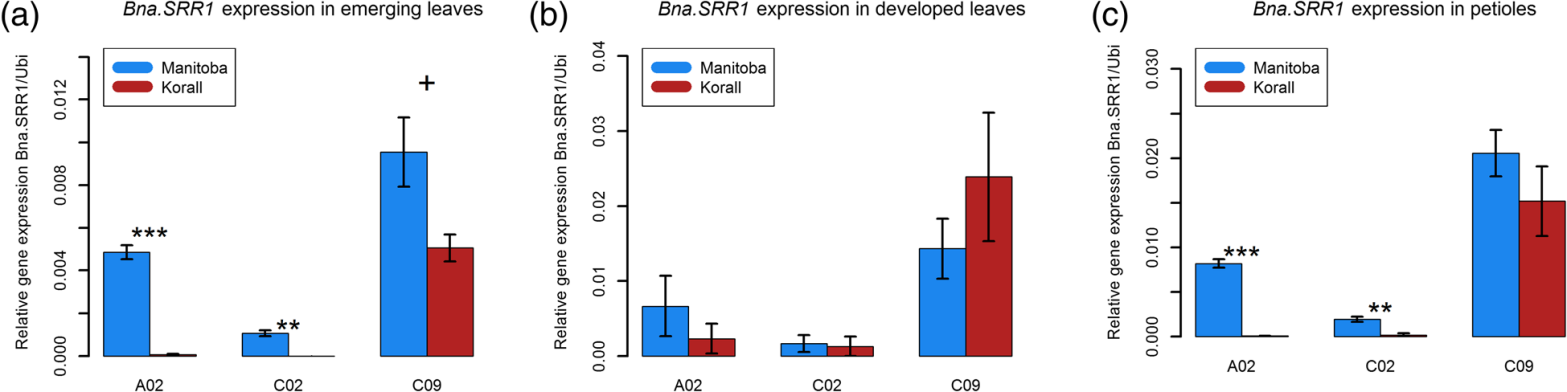
Relative gene expression of the three expressed *Bna.SRR1* copies in emerging and developed leaves and petioles in the accessions Manitoba (winter-type) and Korall (spring-type) without vernalization from rosette material with approximately 5 developed leaves. (**a**) Emerging leaves, (**b**) developed leaves, (**c**) petioles. The values were calculated from RT-qPCR using the ΔCt method and represent mean of 3 biological replicates. Error bars show standard error of mean. Asterisks show the level of significance based on the Student’s t-test (*p-value< 0.05, **p-value< 0.01, ***p-value< 0.001)

**Fig. 3:**
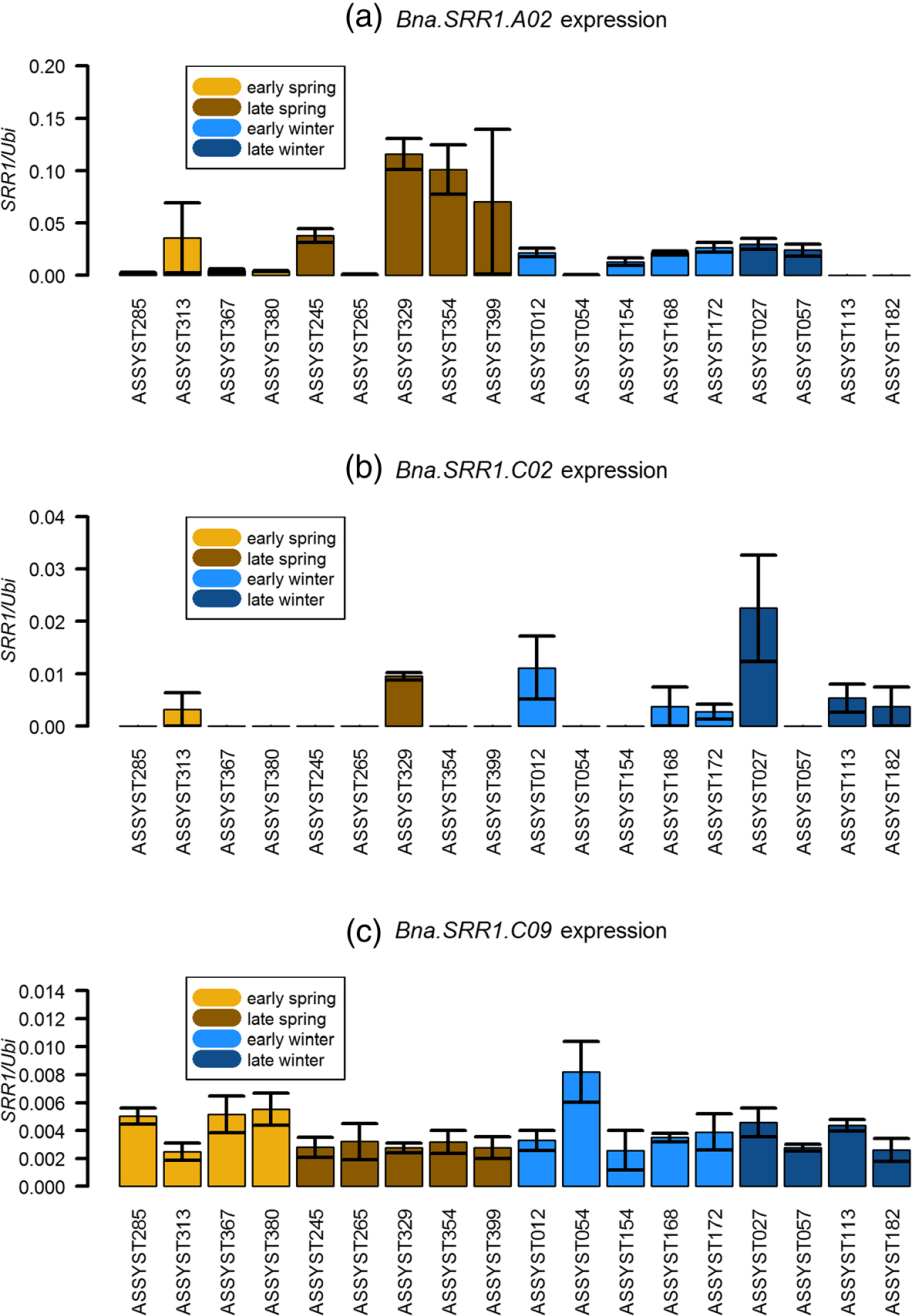
Relative gene expression of *Bna.SRR1* gene copies in early- and late-flowering spring and winter accessions from the ASSYST collection. (**a**) *Bna.SRR1 A02*, (**b**) *Bna.SRR1 C02*, (**c**) *Bna.SRR1 C09*. The values were calculated from RT-qPCR using the ΔCt method and represent mean of 3 biological replicates. Error bars show standard error of mean

By sampling the Manitoba winter type accession, requiring an extended period of cold to be able to flower, and the Korall spring type accession, which does not, potential seasonal differences in expression were examined. For 10 week old plants, emerging leaves, developed leaves and petioles were sampled and expression levels of the different copies were tested in the sampled tissues with RT-qPCR using copy-specific primers. This revealed that only three of the five gene copies were expressed at detectable levels, namely the *A02*, *C02* and the *C09* gene copies (Fig. 2). Of these, the *C09* copy was expressed at higher levels compared to the other gene copies, accumulating to about two times the levels of the *A02* copy in all tested tissues in the Manitoba winter type and to an even higher ratio in the Korall spring type (Fig. 2). The *C02* copy was expressed at lower levels than both the *A02* and *C09*. In emerging leaves, all expressed gene copies were expressed at higher levels in the winter type Manitoba, compared to the spring type Korall (Fig. 2a). In developed leaves, expression levels were more similar between the accessions and the *C09* copy was expressed at a slightly higher, but not significantly, level in the spring type Korall compared to the winter type Manitoba (Fig. 2b). In petioles, expression of the *A02* and *C02* copies was only detectable in the Manitoba winter type while the *C09* copy was expressed at high levels in both Korall and Manitoba (Fig. 2c). Thus, there is a much more prominent difference in expression level between accessions in emerging leaves compared to developed leaves. This may suggest that the *Bna.SRR1* genes have an important regulatory role at an earlier stage of development in the Manitoba winter accession compared to the Korall spring accession. To examine whether these findings were accession-specific or dependent on the winter type vernalization requirement, nine additional winter and spring accessions of the ASSYST collection [42, 43], were sampled for emerging leaf material and the expression of *Bna.SRR1 A02, C02* and *C09* was examined. Five accessions were classified as early flowering and four as late flowering of the winter types, while four accessions were early flowering and five late flowering of the examined spring types. Analysis of these accessions revealed a large variation in expression of the *A02* gene copy between the accessions (Fig. 3a). Interestingly, the late-flowering spring lines had a statistically significant (*p* > 0.01, two-factorial ANOVA) higher expression of the *A02* copy compared to the early-flowering spring lines.

The *C02* gene copy was expressed differently between accessions, expression levels were generally higher in the winter accessions, but in several accessions, no expression at all was detected (Fig. 3b).

Expression of the *C09* copy was more stable between the different accessions and comparable to what was observed in the Korall and Manitoba accessions, suggesting presence of the *C09* gene product is important in both winter and spring types (Fig. 3c). Additionally, to examine *Bna.SRR1* expression in other tissues, roots, stems and flowers were sampled from the spring accession Ability as well as roots and stems from the winter accession Zephir. Expression of *Bna.SRR1* was subsequently tested. No *Bna.SRR1* gene copy could be detected in roots, while expression of A02, *C02* and *C09* was detected in stems (Additional file 1: Figure S1). Here, the *A02* copy was expressed at higher levels than the *C02* and *C09* copies in the winter accession, while the *C09* copy had a similar level of expression in both accessions in stems and in flowers in the spring accession. The copy on *C02* was expressed at similar levels to *C09* in stems in both accessions, but not detectable in flowers. In conclusion, the *A02* and *C09* copies were detected in stem and flower tissue, while the *C02* copy was only detected in stems, suggesting possible tissue-specific subfunctionalization between the gene copies.

### *Bna.SRR1* gene copies show different ability to rescue early flowering in *srr1–1*

**Fig. 4:**
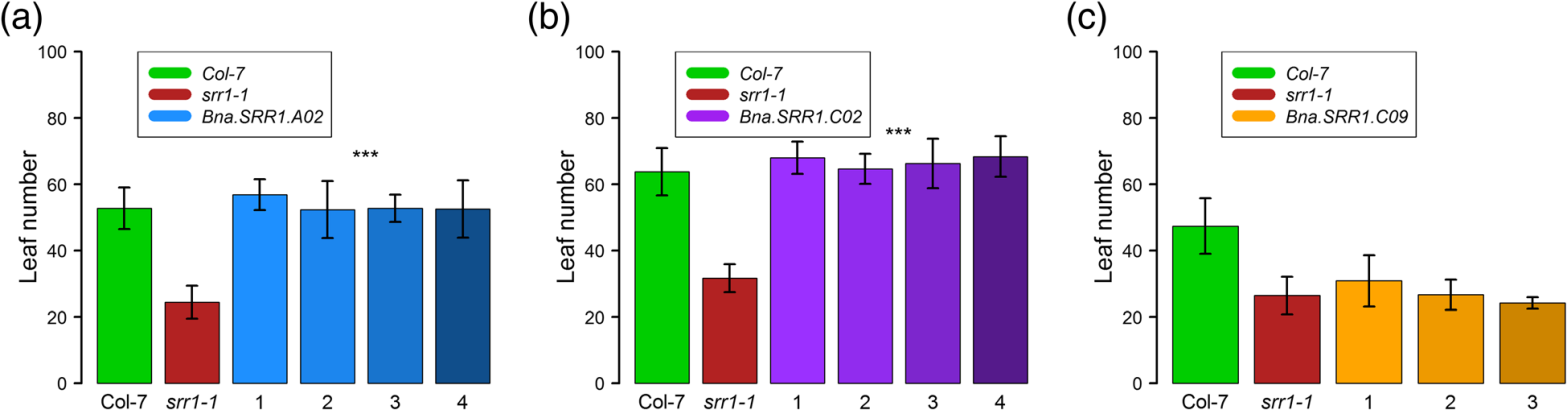
Flowering time of *srr1–1* plants transformed with *Bna.SRR1* gene copies. Plants were grown in SD conditions (16 h light: 8 h dark, 20 °C). (**a**) *Bna.SRR1 A02*-transformed lines, (**b**) *Bna-SRR1 C02*-transformed lines, (**c**) *Bna.SRR1 C09*-transformed lines. Leaves of at least 15 plants per line were counted at flowering. Error bars show standard deviation. Asterisks show the level of significance based on the Student’s t-test compared to not transformed *srr1–1* plants (*p-value< 0.05, **p-value< 0.01, ***p-value< 0.001)

To examine whether the *Bna.SRR1* gene copies may have a similar function in flowering as the Arabidopsis *SRR1* gene, the three gene copies shown to be expressed in *B. napus* (*A02*, *C02* and *C09*) were introduced into *srr1–1* mutant plants. About 1500 bp of the promoter region and the genes including the 3′ untranslated region were amplified from genomic *B. napus* DNA using PCR and introduced into the HPT1 binary vector [44]. Subsequently, *srr1–1* mutant plants were transformed with these vectors to introduce the *Bna.SRR1* copies into Arabidopsis. The transformed plant lines were tested for their flowering phenotype under SDs, where *srr1–1* mutants are known to have a strong early flowering phenotype [1]. Flowering time of the transformed plant lines was then measured. The plants transformed with the *A02* gene copy as well as the *C02* copy flowered similar to Col-7 wt plants, thus fully complementing the early flowering phenotype of *srr1–1* (Fig. 4a, b). In comparison, the plants transformed with the *C09* copy flowered with the same leaf numbers as the *srr1–1* mutants (Fig. 4c). This suggests that the differences in *C09* compared to the other homologs may be critical for the proteins’ ability to repress flowering in Arabidopsis. In contrast, the deletion in *C02* has no relevance for the function of the protein in regulation of flowering.

To examine how the difference in amino acid composition in C09 may alter the protein, the predicted protein structure of the different SRR1 copies was generated using the PredictProtein resource [45]. This showed that the SRR1 homologs are predicted to have a very similar structure (Additional file 2: Figure S2). The major difference in C09 compared to the other copies is that one α-helix, predicted to be mainly exposed, is missing through the deletion. The prediction does not suggest that the deletion renders the protein unstable.

### Expression of *Bna.SRR1* gene copies in Arabidopsis

As expression levels of the different *Bna.SRR1* gene copies differed strongly in *B. napus*, the level of expression of *Bna.SRR1.A02*, which could complement flowering in Arabidopsis, and *C09*, which could not, was tested in the Arabidopsis lines transformed with the respective gene copies.

**Fig. 5:**
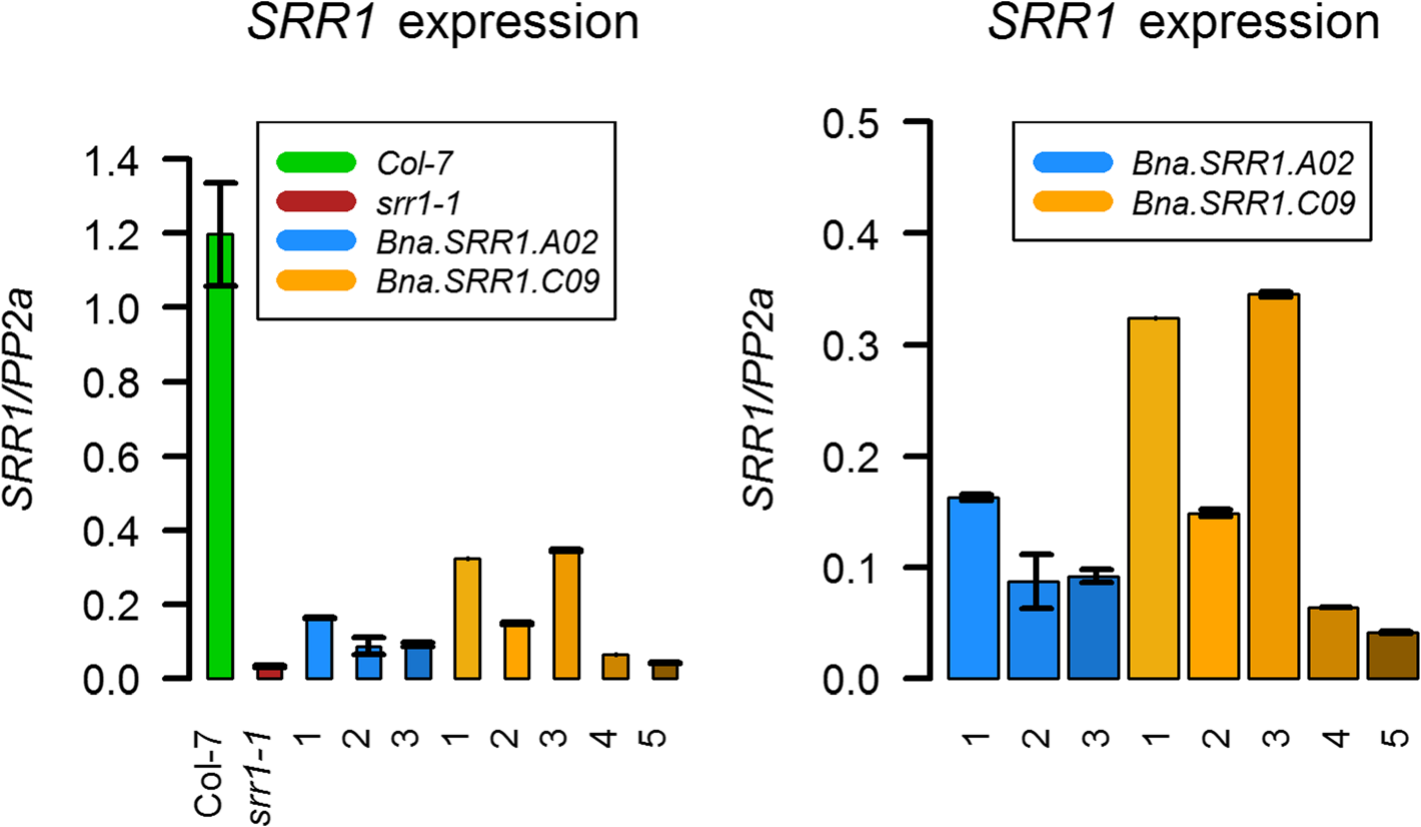
Expression of *SRR1* in Col-7 wt and *srr1–1* lines transformed with the *Bna.SRR1* gene copies. Left panel: Expression in comparison with Col-7 wt of three independent transformed lines transformed with *Bna.SRR1 A02* and *Bna.SRR1 C09*, respectively. Right panel: magnified comparison of expression between the *Bna.SRR1 A02*-transformed and *Bna.SRR1 C09*-transformed lines. Error bars show standard error of mean

RT-qPCR analysis showed that in comparison to the endogenous *SRR1* gene copy, both *Bna.SRR1* genes introduced into the *srr1–1* background were expressed at lower levels (Fig. 5). For the *A02* copy, these low levels of expression were obviously sufficient to complement the flowering phenotype. The *C09* copy was also expressed at lower levels than *AtSRR1*, but higher than *A02* in the tested lines, reaching ca 30% of expression levels of *AtSRR1*. The level of expression of the *A02* copy does not appear to be critical for the function of SRR1, as low amounts of transcript are sufficient to fulfill its role in flowering time control. A comparison of the promoter structure between the *SRR1* gene copies using the MEME suite [46] revealed two enriched motifs common in all the gene promoters, although their distribution is somewhat different between the genes (Additional file 3: Fig. S3). The motifs, a SORLIP motif and an ARF motif, have been described to be involved in light-regulated gene expression and as an auxin response factor binding site, respectively [47, 48]. They were located close to the start of the coding sequence in *AtSRR1*, while they were located further upstream in the *Bna.SRR1* gene promoters. Although the factors regulating SRR1 expression are unknown, this may indicate that the efficiency of transcriptional activation of the *Bna.SRR1* genes in Arabidopsis is different, which could explain the reduced expression levels of the *Bna*.*SRR1* copies compared to endogenous *AtSRR1*.

### Expression of SRR1 targets in *Bna.SRR1* transformed lines

SRR1 acts in several pathways regulating flowering by promoting the expression of *FT* repressors [1]. To examine how the *Bna.SRR1* copies affected known targets of AtSRR1 in regulation of gene expression, their transcript levels in plants carrying the *AtSRR1*-complementing *A02* and the non-complementing *C09* gene copies were measured.

**Fig. 6:**
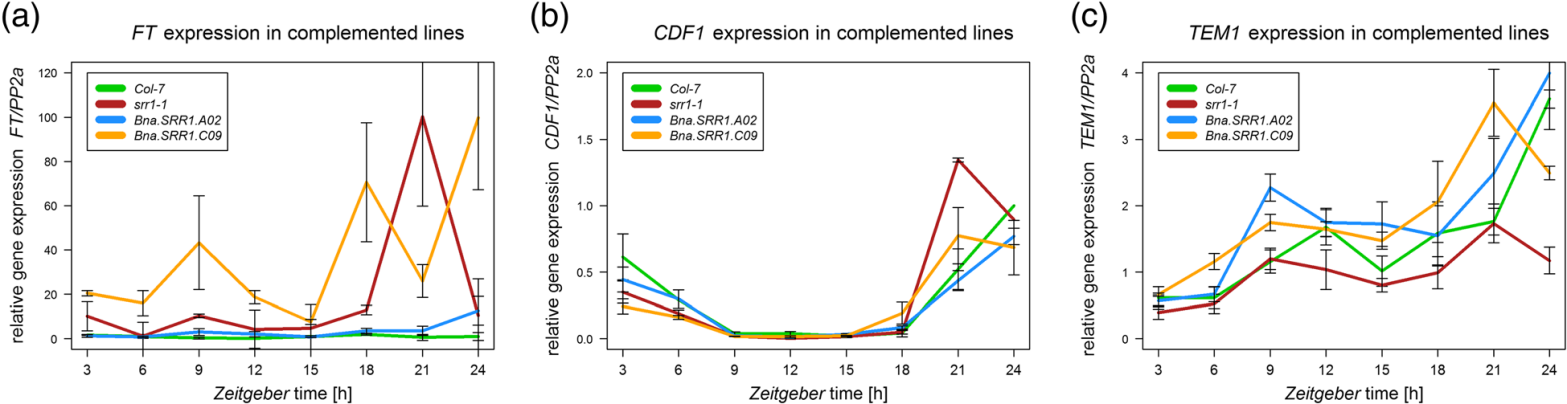
Relative expression of known SRR1 targets in lines transformed with *Bna.SRR1 A02* and *Bna.SRR1.C09*. (**a**) *FT,* (**b**) *CDF1,* (**c**) *TEM1.* The values represent the average expression of three independently transformed lines. Error bars show standard error of mean

To confirm that the complemented phenotype in *BnaSRR1.A02* lines was due to restoration of the *FT* expression pattern, a time series was sampled in 3-h intervals over 24-h in SD conditions and analyzed using RT-qPCR. This revealed that in *A02*-transformed lines, *FT* was expressed at very low wt-like levels, while elevated expression was observed in *C09*-transformed lines, notably at the for flowering induction critical time point ZT9, as well as in *srr1–1* mutants (Fig. 6a, Additional file 4: Fig. S4). Furthermore, analysis of *CDF1* expression, a known repressor of *FT* and a target of SRR1 showed that *CDF1*, with an expected peak of expression in the morning, was expressed as in Col-7 in the lines transformed with the *A02* gene copy. Meanwhile, *CDF1* was expressed at reduced levels in the morning and expression peaked earlier in the *C09*-transformed lines (Fig. 6b). This was similar to the expression pattern observed in *srr1–1* mutants and thus *C09* had no complementing effect on *CDF1* expression.

*TEM1* and *TEM2* transcription factors are other known targets of SRR1, which are involved in regulation of flowering through the GA biosynthesis pathway [49]. Due to their redundancy and similar expression, *TEM1* was examined to determine whether the *Bna.SRR1* copies could affect their expression. Interestingly, whereas *srr1–1* showed reduced expression, as seen previously [1], both the *A02* and *C09*-transformed lines showed an expression pattern similar or even slightly enhanced compared to Col-7 wt, suggesting complementation of *TEM1* expression (Fig. 6c). TEM1 is known to repress the gibberellic acid biosynthesis gene *GIBBERELLIN 3-OXIDASE 1* (*GA3OX1)* [49]. To confirm the rescued expression of *TEM1* in *A02* and *C09*-transformed lines, *GA3OX1* expression was measured. Transcript levels were elevated in *srr1–1* compared to wt, congruent with previous observations [1]. In the *C09*-transformed lines no significant difference was seen while expression in *A02*-transformed lines was somewhat reduced (Additional file 5: Figure S5). The results support the elevated *TEM1* levels in the transformed lines. In conclusion, this suggests that the function of SRR1 in transcriptional regulation is fully rescued by the *A02* gene copy for all tested genes, while the *C09* gene copy can only complement *TEM1* expression, which is not enough to rescue the early flowering phenotype of *srr1–1*.

## Discussion

Our data show that SRR1 is highly conserved in *Brassicaceae,* suggesting an important function in growth and development within this family (Fig. 1a). However, its unique protein structure has made the prediction of key regions of the protein or a molecular mode of action difficult. Interestingly, we found that the crop species *Brassica napus* carries in total five homologs with differences in gene and protein structure between them, including a larger deletion in the A03, A10 and C09 proteins, compared to the A02 and C02 proteins and AtSRR1 (Fig. 1b). As this could suggest subfunctionalization between the different gene copies, we tested their level of expression in *B. napus* followed by a functional analysis of the expressed copies in Arabidopsis *srr1–1* background, where *AtSRR1* is not expressed.

### Differential gene expression suggests subfunctionalization

Initial gene expression analysis in the *B. napus* accessions Manitoba (winter type) and Korall (spring type) showed that only three of the five gene copies were expressed, *A02*, *C02* and *C09* (Fig. 2). Expression of the same copies was also detected in stems, while in flowers only *A02* and *C09* was detected (Additional file 1: Figure S1). In roots, no *Bna.SRR1* copy was detected.

Differential expression of *B. napus* flowering gene copies has been shown in several reports [25, 50–52]. Here, the *C09* copy is consistently expressed in all tested accessions and tissues, while the *A02* and C02 copies are expressed at different levels depending on accession in emerging leaves (Fig. 3).

In the winter type Manitoba and the spring type Korall, comparison of expression between developed and emerging leaves showed that differences in expression between accessions was lower in comparison to emerging leaves (Fig. 2a, b), suggesting that the *A02* and *C02* gene copies may have a repressive role on flowering at early stages of development, when highly expressed. This may suggest that they have a comparable role as SRR1 in Arabidopsis, in suppressing flowering until the conditions are more favorable. Interestingly, a similar pattern has been observed for the important flowering time regulator, *FLC*, where three out of nine copies were differentially expressed between winter and spring material (Quezada et al., submitted). One copy of *Bna.FLC* was never expressed [53], similar to what we found for *Bna.SRR1.A03* and *Bna.SRR1.A10*, indicating pseudogenization.

Thus, in Manitoba and Korall, the larger difference in *Bna.SRR1* expression in emerging leaves compared to developed leaves between the Korall spring and the Manitoba winter type may suggest that high expression early in the developmental cycle in the winter type is desirable to prevent premature flowering. This could account for a mechanism measuring the ratio of developing to differentiated leaves, allowing flowering only after a certain leaf mass has been reached. Developing leaves could likewise send a “stop” signal, which is only overridden if enough differentiated tissue has developed.

However, in the extended analysis of emerging leaves of several other accessions, *A02* expression displayed a large variation suggesting that such a mechanism may be accession-dependent. *A02* was particularly highly expressed in several late-flowering spring accessions, suggesting it may have a role in delaying flowering in these accessions (Fig. 3a). This function may be overruled by *FLC* in winter accessions with a vernalization requirement.

Expression of *C02* also varied between accessions, suggesting a possible accession-specific function, while expression of *C09* was much more stable between accessions in the extended analysis (Fig. 3b, c). In comparison, presence of the *C09* gene product seems to be of general importance in *B napus* and thus have the different gene copies subfunctionalized to perform specific roles in this species.

### Complementation reveals potential key protein domain of SRR1

Flowering time experiments with the three gene copies being expressed in *B. napus* showed that only the *A02* and *C02* gene copies can complement the early flowering phenotype of *srr1–1* while the *C09* copy cannot (Fig. 4). This suggests that the differences in *C09* may be critical for the function of the SRR1 protein in Arabidopsis, at least in regard to its role in regulating flowering. The most obvious candidate region to be critical for correct function is the 21 amino acid deletion in C09. In comparison, although the C02 protein product also carries a deletion in another part of the protein, it could still complement the loss of AtSRR1 in *srr1–1* plants (Fig. 1b, Fig. 4). As the SRR1 protein sequence does not contain any known regulatory elements, this is an important finding, indicating that this region of the protein may be critical for proper function. This deletion is a highly conserved SRR1-unique sequence in *Brassicacea* and this specific deletion only occurs in *B. napus*. Taking into account that the *A02* and *C02* copies are the same ones expressed at much lower levels in the spring type compared to the winter type, this further indicates that their expression may be necessary to prevent undesirable premature flowering in the winter type, acting as a repressive signal in months preceding the cold season.

The dysfunction of the *C09* gene copy in Arabidopsis could be either due to an important function-specific binding region of the protein being excluded through the altered protein sequence, or due to direct degradation of the C09 protein product. However, the performed protein structure prediction suggests that C09 still has a similar structure to the other SRR1 copies, and only one predicted helix structure is missing (Additional file 2: Figure S2). Considering the experimental results, this deletion may be important for interactions or protein modifications necessary for the regulation of flowering time. Further biochemical studies are however necessary to confirm that the region deleted in C09 is the determining factor.

Considering that SRR1 in Arabidopsis is also involved in circadian regulation and light signaling [37], it is possible that the *A02* and *C09* copy may have a specialized functions in *B. napus,* through subfunctionalization. Being that the *C09* gene copy is unique for *B. napus* may also suggest a species-specific specialization. Its exact function requires more detailed analyses in *B. napus*.

The expression analysis in lines complemented with *B. napus* gene copies in Arabidopsis shows that the expression levels of the introduced genes were much lower than the endogenous *SRR1* in Col-7 wt plants (Fig. 5). This was, however, sufficient for *A02* to be able to complement the *srr1–1* early flowering phenotype, suggesting that low *SRR1* expression levels are enough for proper function. Expression of the *C09* copy was lower than endogenous *SRR1*, but higher than *Bna.SRR1.A02*. Thus, considering that expression of the *A02* lines was sufficient to complement the flowering phenotype of *srr1–1*, it is unlikely that the level of *C09* expression is a major factor in the inability of the *C09* gene copy to do the same (Fig. 5).

### *CDF1* is key to regulate flowering through SRR1

Analysis of known targets of SRR1 showed that the *A02* gene copy was able to replace AtSRR1 function in regard to its role in regulating expression of flowering time regulators, including the key florigen *FT,* the important *FT* repressor, *CDF1,* and *TEM1* (Fig. 6). In contrast, the *C09* copy was unable to rescue *SRR1* function, as the *C09*-transformed lines showed *srr1–1*-like expression patterns of *CDF1* and *FT*. Conversely, *TEM1* expression levels were rescued to WT levels by *C09*, but this seems to have a limited effect on flowering, as *C09*-transformed plants flowered like *srr1–1* mutants. In conclusion, the data suggests that the key target for floral repression by SRR1 is *CDF1,* where an altered expression is observed in *srr1–1,* as well as in *C09*-transformed lines (Fig. 6a). *TEM1* appears to be rescued by both the *A02* and *C09* gene copies (Fig. 6c), although this is not enough to rescue the early flowering phenotype in *C09*-transformed lines. This indicates that the differences in C09, most notably the deleted region, may be necessary for SRR1 control of *CDF1* expression.

Our data suggests that these gene copies may have a similar molecular mode of action in *B. napus* as in Arabidopsis and may be able to influence expression of *B. napus* homologs to other known flowering time components, which have been shown to be also present in *B. napus* [33]. Furthermore, the consistent expression levels of the *C09* copy compared to the variation in *A02* expression may suggest that the gene copies have subfunctionalized to acquire specific roles in *B. napus* that may or may not be related to the regulation of flowering. This information may help to map the signaling network controlling flowering time in *B. napus*, enabling the identification of key factors in breeding.

## Conclusions

We have shown that SRR1, an important Arabidopsis flowering time regulator, has several homologs in *Brassica napus*. Their expression patterns varied and major alterations in amino acid composition were found. The differences in expression between winter and spring type accessions suggest their expression may be of importance to flowering ability.

Only two of three expressed copies could complement the early flowering *srr1–1* mutant phenotype, showing cross-species functionality. The C09 copy, with a 21 amino acid deletion compared to A02, C02, and AtSRR1, failed to complement the early flowering phenotype. *C09* is, however, consistently expressed in *B. napus*, suggesting strong subfunctionalization between the gene copies. The presented data may be used in the future for further characterization of the flowering time pathway in *B. napus* and highlights the possibility that the *B. napus* gene copies may have taken on specific functions throughout evolution.

## Methods

### Sequence analysis

Whole genome sequences for *A. thaliana, A. lyrata, B. napus, B. rapa, B.oleracea, Camelina sativa, Capsella rubella, Thelungiella salsunginea, Thelungiella halophila, Aethionema arabicum, Leavenworthia alabamicum, Schrenkiella parvula* and *Sisymbrium irio* were retrieved from http://brassicadb.org/brad/ftpTrans.php. The five known copies of *B. napus* were then used for a BLAST search against each of the genomes. *Bna*.*SRR1* copies were then selected using a cut-off value of 10^− 50^ for Brassica and Arabidopsis, while using a cutoff of 10^− 20^ for the remaining species. Fragments shorter than 200 bp were excluded. To avoid missing gene information, 100 bp were added to the start and stop of each BLAST position. For all species except the *Brassica* species and *Arabidopsis thaliana*, peptide sequences were predicted using GENSCAN (http://genes.mit.edu/GENSCAN.html) with “Arabidopsis” as organism. For *Brassica* and *A. thaliana*, we used the peptide sequence information from the respective peptide prediction published within their reference genomes.

### Plant material and growth conditions

#### *Arabidopsis thaliana*

The T-DNA mutant *srr1–1* in the Col-7 background has been described [1, 37]. All seeds were stratified for 3 d at 4 °C before putting on soil. Seeds grown on plates were surface sterilized and stratified for 3 d at 4 °C before plating on agar-solidified half-strength MS (Murashige & Skoog) medium (Duchefa) supplemented with 0.5% sucrose and 0.5 g MES. Plants were grown in Percival incubators AR66-L3 (CLF Laboratories) in 100 μmol m^− 2^ s^− 1^ light intensity, with the light-dark and temperature conditions as indicated.

#### *Brassica napus*

A winter accession (Manitoba) and a spring accessions (Korall) of oilseed rape were sown in 7 × 7 cm pots in 3 biological replicates and transplanted to 12 × 12 cm pots 4 weeks after sowing. For the extended expression analysis, a diversity set consisting of 10 winter and 10 spring accessions was sown in quickplates in 3 biological replicates. Cultivation was performed in a greenhouse using a 16 h/8 h day/night rhythm with 20 °C /17 °C. For Manitoba and Korall, we sampled petioles, developed and emerging leaves separately 10 weeks after sowing. For the diversity set, we selected 9 winter and 9 spring accessions for the youngest developed leaf 8 weeks after sowing. The other two accessions were grown 3 weeks further and we sampled stems, roots and flowers separately. Tissues were frozen in liquid nitrogen and stored at − 80 °C until RNA extraction.

### Flowering time experiments

Seeds were germinated as described above and grown on soil in a random fashion. Flowering time was determined by counting the rosette leaves when the bolt was > 0.5 cm tall [54].

### Cloning

Genomic DNA from *Brassica napus* was amplified using Phusion Proofreading polymerase (Thermo Fischer) and primers with specific restriction sites. The amplified DNA was separated on an agarose gel and extracted using a GeneJet gel extraction kit (Thermo Fischer) and then ligated into a pJET2.1 cloning vector using the CloneJet kit (Thermo Fischer). The insert was digested and separated on an agarose gel and then cloned into a pHPT1 binary vector [44], using T4 Ligase (Thermo Fischer). The resulting construct was transformed into Agrobacterium and then into Arabidopsis *srr1–1* plants using the floral dip method.

### Transcript analysis

#### Arabidopsis material

Total RNA was extracted using from plant material using Tri Reagent as previously described or using Universal RNA Purification Kit (Roboklon) following manufacturer’s instructions.

For cDNA, 2 μg of total RNA was DNAse-treated using RQ1 RNAse-free DNAse (Promega) and reverse transcribed using AMV Reverse Transcripase (Roboklon) according to manufacturer’s instructions.

qPCR was performed with iTaq Sybr Green Supermix (Bio-Rad) according to manufacturer’s instructions. The normalized expression level was determined using the ΔCt method, with *PP2a* (At1g69960) as a reference gene as described [55]. The primer sequences can be found in Additional file 6: Table S1.

#### *Brassica napus* material

Total RNA was extracted using the NucleoSpin miRNA kit (Macherey-Nagel) following manufacturer’s instructions. The eluted RNA was quantified using Qubit RNA Broad Range on a Qubit fluorimeter and stored at − 80 °C until use.

Primers were designed based on the Darmor-bzh reference genome, version 4.1 (Chalhoub et al. 2014). Specificity was confirmed by aligning the predicted cDNA with CLUSTAL multiple sequence alignment by MUSCLE (http://www.ebi.ac.uk/Tools/msa/muscle/, version 3.8). The primer sequences can be found in Additional file 6: Table S1.

cDNA synthesis was performed using the RevertAid cDNA synthesis kit (ThermoFisher) using 1 μg of total RNA and Oligo-dT primers. The amount of cDNA was quantified using the Qubit DNA High Sensitivity kit on a Qubit fluorimeter. Quantitative Real-time PCR was performed on a Real-Time PCR System ViiA7 cycler (Applied Biosystems) in 384 well plates. The reaction mix containing specific primers, the template cDNA and FastStart Universal SYBR Green Master mix containing Rox (Roche) was pipetted by a robot (Biomek 4000, Beckman Coulter). As endogenous control, we used ubiquitin. The PCR program was as follows: initial denaturation (94 °C for 2 min), amplification and quantification (40 cycles, 95 °C for 20 s, 60 °C for 30 s, 72 °C for 30 s), and a final extension (72 °C for 5 min). At the end, a melting curve was recorded between 55 and 95 °C. PCR efficiency was measured using a pool of all samples in a dilution series of 6 points. All samples were measured in 3 technical replicates. The normalized expression level was determined using the ΔCt method.

## Additional files

Additional file 1: **Figure S1.** Relative gene expression of *Bna.SRR1* gene copies in different tissues of the Ability spring and Zephir winter accessions. The values were calculated from RT-qPCR using the ΔCt method and represent mean of 3 biological replicates. Error bars show standard error of mean. (TIF 10547 kb)

Additional file 2: **Figure S2.** Protein structure predictions based on the PredictProtein server. Red squares in the first row indicate predicted alpha-helices, blue squares indicate strands. Yellow boxes in the second row indicate buried regions while blue boxes indicate exposed regions. Grey boxes in the third row indicate disordered regions. The red dotted squares highlight a predicted helix missing in Bna.C09 compared to the other predicted SRR1 copies. (TIF 517 kb)

Additional file 3: **Figure S3.** *AtSRR1* and *BnSRR1* promoter alignment. Two enriched motifs were discovered using MEME. Sequences from *A.thaliana* and *B.napus* 1 kb upstream from the transcriptional start site were used with a minimal motif length of 6 and maximum of 10 (Bailey and Elkan, Proc Int Conf Intell Syst Mol Biol, 2:28–36,1994). Motifs were determined to be statistically significant with an E-value lower than 0.05. SORLIP 2 binding site is associated with *PhyA* signaling, while ARF (Auxin Response Factor) binding sites are intrinsic for the auxin response. Enriched motifs are underlined and binding sites are highlighted in gray. (TIF 577 kb)

Additional file 4: **Figure S4.** Expression of FT at zeitgeber time 9 (9 h after lights on, ZT9) in plants grown in SDs (8 h light:16 h dark, 20 °C). The values represent biological replicates of three independently transformed lines. Error bars show standard error of mean. (TIF 5273 kb)

Additional file 5: **Figure S5.** Expression of TEM1 target *GA3OX1* at zeitgeber time 8 (8 h after lights on, ZT8) in plants grown in SDs (8 h light:16 h dark, 20 °C). The values represent biological replicates of three independently transformed lines. Error bars show standard error of mean. Asterisks show the level of significance based on the Student’s t-test compared to Col-7 wt plants. (TIF 6591 kb)

Additional file 6: Primer sequences. (XLSX 10 kb)

## Data Availability

All data generated or analysed during this study are included in this published article and its supplementary information files.

## Abbreviations

LD: Long day
RT-qPCR: Real Time Quantitative PCR
SD: Short day
