# Supplementary figures and images for: Different copies of SENSITIVITY TO RED LIGHT REDUCED 1 show strong subfunctionalization in Brassica napus
Source: BMC Plant Biol. 2019 Aug 22;19:372. doi: 10.1186/s12870-019-1973-x (PMC6704554; doi:10.1186/s12870-019-1973-x)

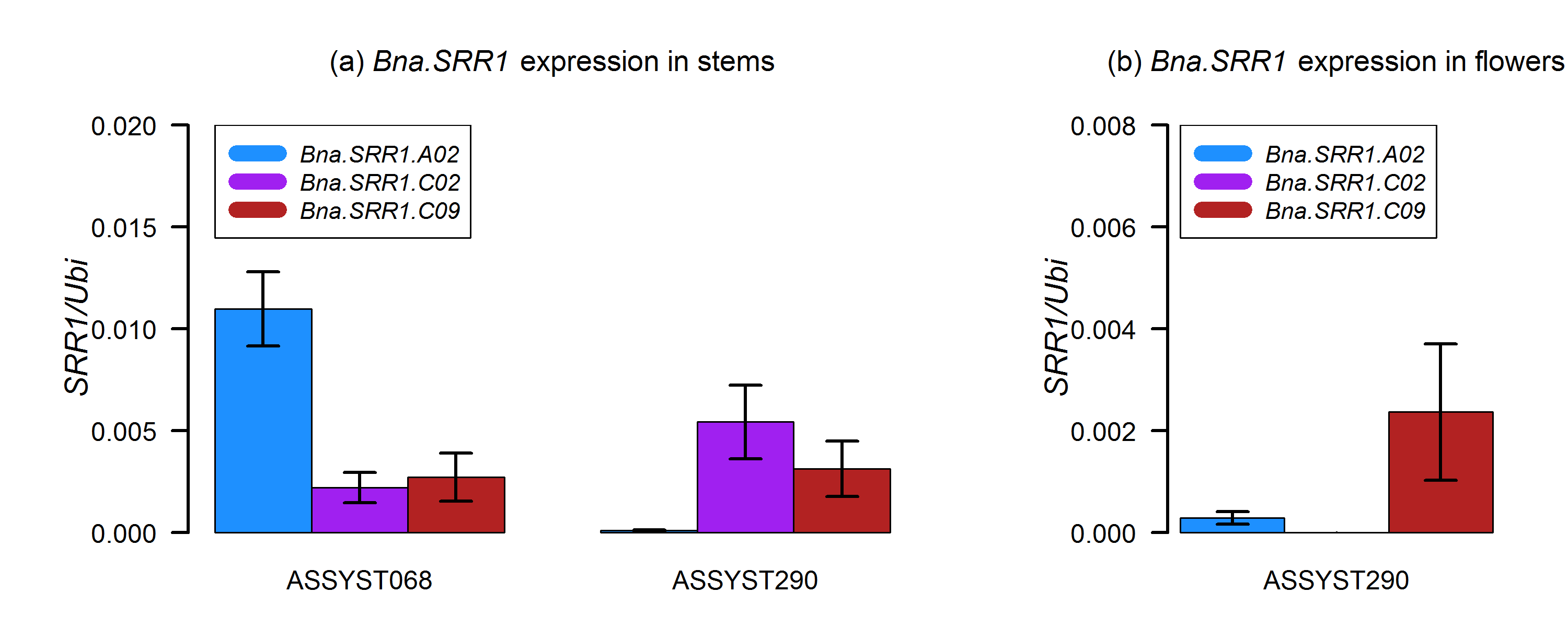

Supplement: Supplementary file 1 — Figure S1. Relative gene expression of Bna.SRR1 gene copies in different tissues of the Ability spring and Zephir winter accessions. The values were calculated from RT-qPCR using the ΔCt method and represent mean of 3 biological replicates. Error bars show standard error of mean. (TIF 10547 kb) [file 12870_2019_1973_MOESM1_ESM.tif]

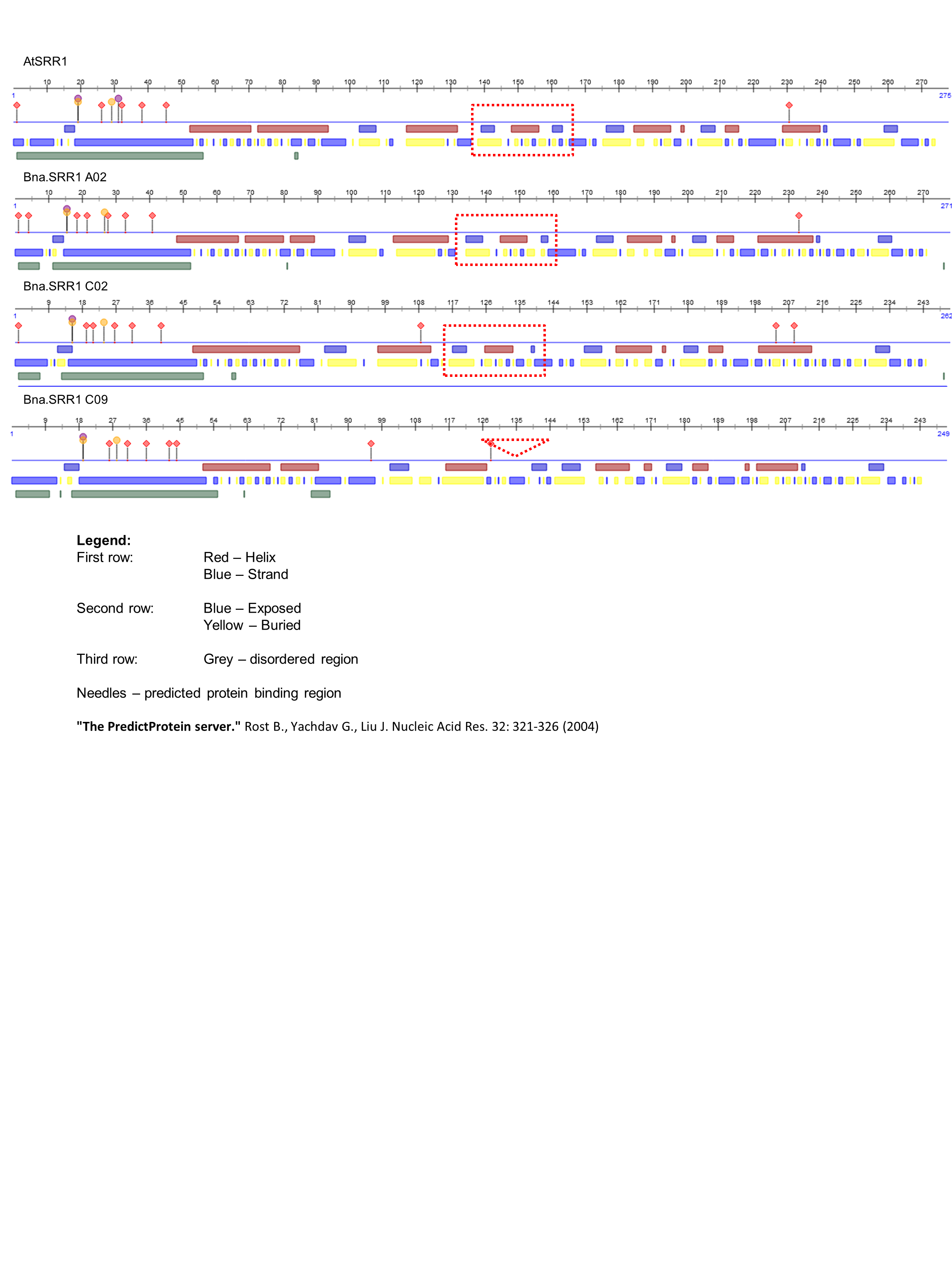

Supplement: Supplementary file 2 — Figure S2. Protein structure predictions based on the PredictProtein server. Red squares in the first row indicate predicted alpha-helices, blue squares indicate strands. Yellow boxes in the second row indicate buried regions while blue boxes indicate exposed regions. Grey boxes in the third row indicate disordered regions. The red dotted squares highlight a predicted helix missing in Bna.C09 compared to the other predicted SRR1 copies. (TIF 517 kb) [file 12870_2019_1973_MOESM2_ESM.tif]

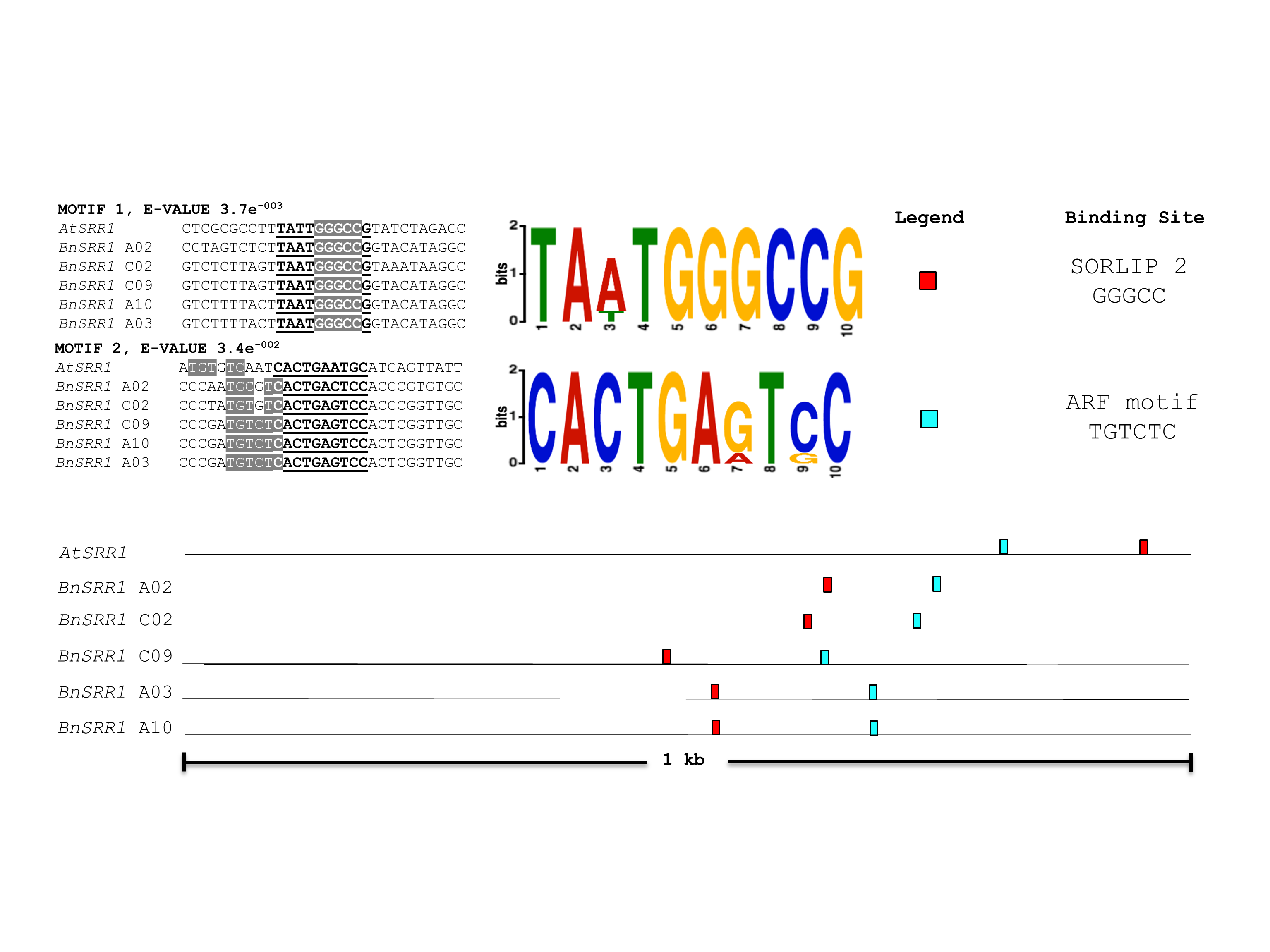

Supplement: Supplementary file 3 — Figure S3. AtSRR1 and BnSRR1 promoter alignment. Two enriched motifs were discovered using MEME. Sequences from A.thaliana and B.napus 1 kb upstream from the transcriptional start site were used with a minimal motif length of 6 and maximum of 10 (Bailey and Elkan, Proc Int Conf Intell Syst Mol Biol, 2:28–36,1994). Motifs were determined to be statistically significant with an E-value lower than 0.05. SORLIP 2 binding site is associated with PhyA signaling, while ARF (Auxin Response Factor) binding sites are intrinsic for the auxin response. Enriched motifs are underlined and binding sites are highlighted in gray. (TIF 577 kb) [file 12870_2019_1973_MOESM3_ESM.tif]

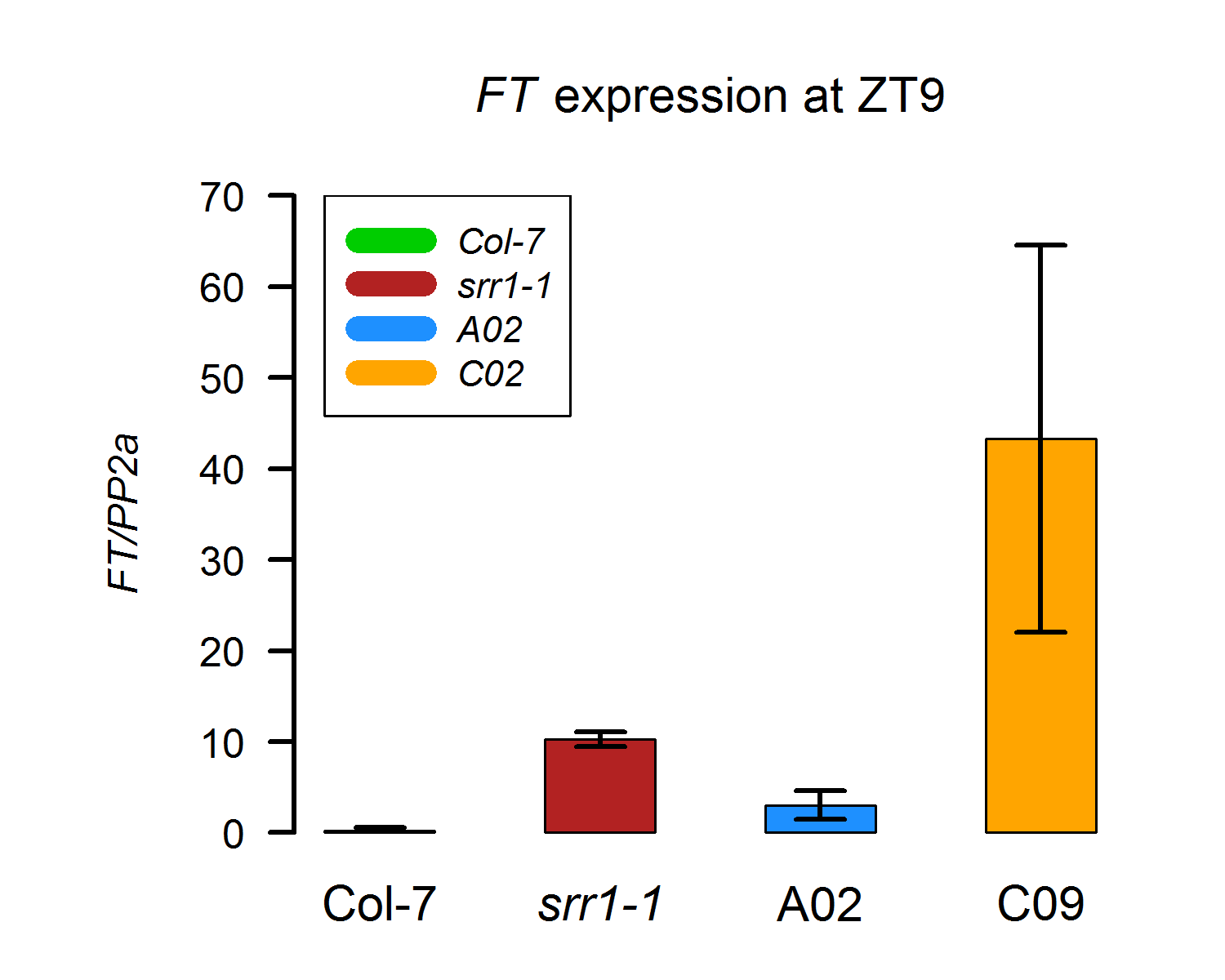

Supplement: Supplementary file 4 — Figure S4. Expression of FT at zeitgeber time 9 (9 h after lights on, ZT9) in plants grown in SDs (8 h light:16 h dark, 20 °C). The values represent biological replicates of three independently transformed lines. Error bars show standard error of mean. (TIF 5273 kb) [file 12870_2019_1973_MOESM4_ESM.tif]

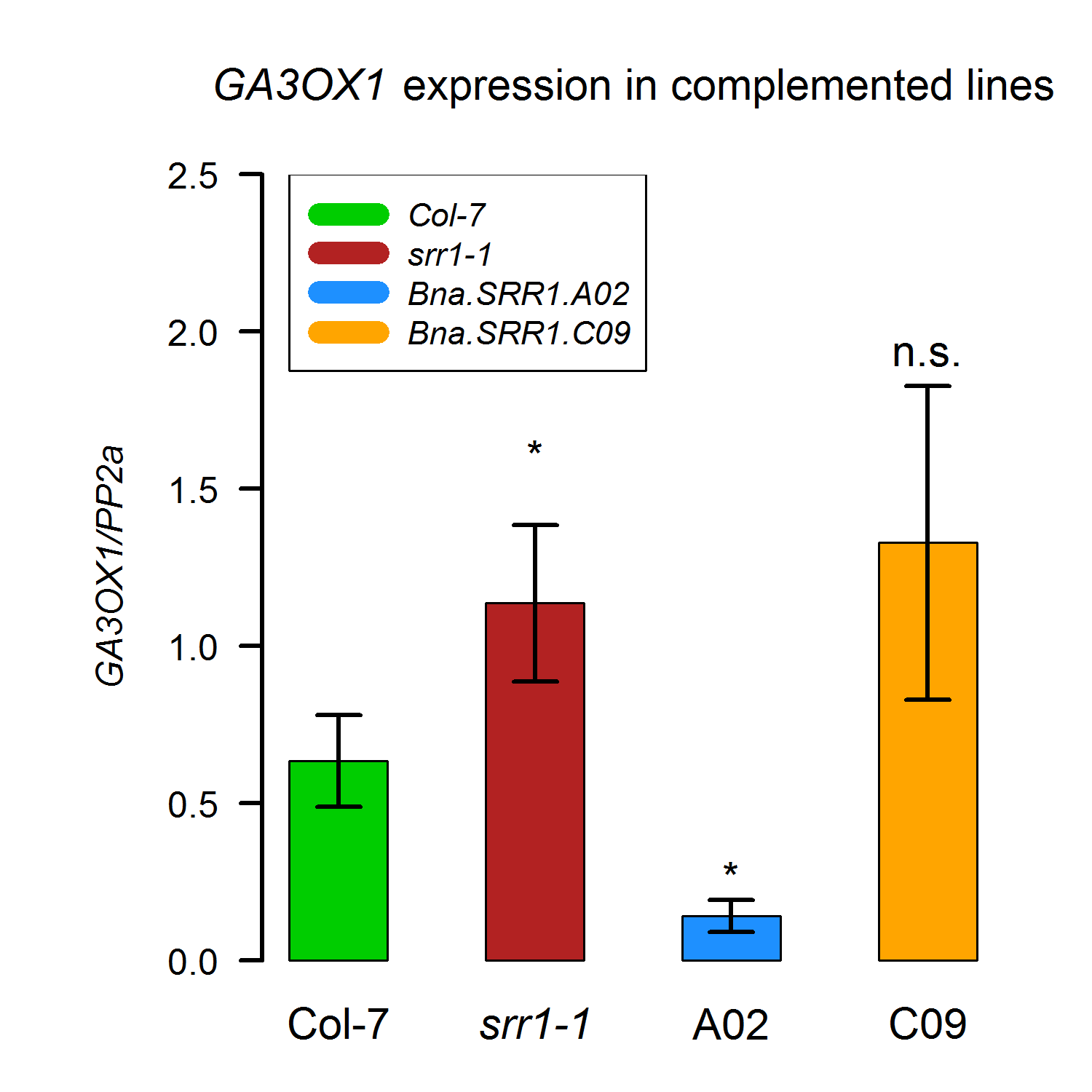

Supplement: Supplementary file 5 — Figure S5. Expression of TEM1 target GA3OX1 at zeitgeber time 8 (8 h after lights on, ZT8) in plants grown in SDs (8 h light:16 h dark, 20 °C). The values represent biological replicates of three independently transformed lines. Error bars show standard error of mean. Asterisks show the level of significance based on the Student’s t-test compared to Col-7 wt plants. (TIF 6591 kb) [file 12870_2019_1973_MOESM5_ESM.tif]
